# Solidagenone from *Solidago chilensis* Meyen Protects against Acute Peritonitis and Lipopolysaccharide-Induced Shock by Regulating NF-κB Signaling Pathway

**DOI:** 10.3390/ph17030273

**Published:** 2024-02-21

**Authors:** Ivanilson Pimenta Santos, Laís Peres Silva, Dahara Keyse Carvalho Silva, Bruna Padilha Zurita Claro dos Reis, Temistocles Barroso de Oliveira, Andressa Maia Kelly, Edivaldo dos Santos Rodrigues, Claudia Valeria Campos de Souza, José Fernando Oliveira-Costa, Simone Sacramento Valverde, Osvaldo Andrade Santos-Filho, Milena Botelho Pereira Soares, Cássio Santana Meira

**Affiliations:** 1 Gonçalo Moniz Institute, Oswaldo Cruz Foundation, FIOCRUZ, Salvador 40296-710, BA, Brazil; 2 Pharmaceutical Technology Institute—FarManguinhos, Natural Products Department, Oswaldo Cruz Foundation, FIOCRUZ, Rio de Janeiro 21041-250, RJ, Brazilandressa.kelly@far.fiocruz.br (A.M.K.);; 3 Laboratory of Molecular Modeling and Computational Structural Biology, Natural Products Research Institute, Health Sciences Center, Federal University of Rio de Janeiro, Rio de Janeiro 21941-599, RJ, Brazilclaudiavaleria@ippn.ufrj.br (C.V.C.d.S.); osvaldo@ippn.ufrj.br (O.A.S.-F.); 4 Center for Infusions and Specialized Medicines of Bahia, Bahia State Health Department, Salvador 41745-900, BA, Brazil; josefernandocosta@hotmail.com; 5 Institute of Innovation in Advanced Health Systems (ISI SAS), University Center SENAI/CIMATEC, Salvador 41650-010, BA, Brazil

**Keywords:** solidagenone, inflammation, endotoxic shock, NF-κB

## Abstract

Anti-inflammatory agents are widely used for the treatment of inflammatory diseases. Nevertheless, the associated side effects of the available drugs make it necessary to search for new anti-inflammatory drugs. Here, we investigated the anti-inflammatory activity of solidagenone. Initially, we observed that a single dose of 30, 60, or 90 mg/kg of solidagenone did not result in mortality or elicit any discernible signs of toxicity in mice. At the same doses, solidagenone promoted a significant reduction in the migration of neutrophils in an acute peritonitis model and decreased mortality in a lipopolysaccharide-induced endotoxic shock model. Interestingly, treatment with solidagenone conferred a protective effect against leukopenia and thrombocytopenia, hematological disorders commonly observed in sepsis conditions. In addition, treatment with all the doses of solidagenone promoted a significant reduction in nitric oxide, TNF-α, and IL-1β levels relative to the LPS-stimulated vehicle-treated cultures. Furthermore, gene expression and in silico analyses also supported the modulation of the NF-*κ*B pathway by solidagenone. Finally, in silico pharmacokinetics predictions indicated a favorable drugability profile for solidagenone. Taken together, the findings of the present investigation show that solidagenone exhibits significant anti-inflammatory properties in acute experimental models, potentially through the modulation of the NF-κB signaling pathway.

## 1. Introduction

Inflammation has a pivotal role in rectifying imbalances to the body’s homeostasis and is crucial for the repair, remodeling, and renewal of various tissues under diverse and adverse conditions [1]. Functioning as the primary line of defense, inflammation safeguards the host from infections induced by a spectrum of pathogens, including bacteria, fungi, parasites, and viruses [2]. Additionally, other stimuli, such as cellular damage, chemical agents, physical injuries, burns, radiation, freezing, ischemia, and reperfusion, can incite inflammatory responses [3].

Classically, the treatment of inflammation entails the administration of non-steroidal anti-inflammatory drugs or glucocorticoids [4,5]. Both exhibit varying degrees of effectiveness in alleviating inflammation, and their use is accompanied by a spectrum of adverse effects such as atherosclerosis, cardiac alterations, gastrointestinal disorders, hypertension, renal toxicity, type 2 diabetes, visceral obesity, and others [5,6].

Therefore, the development of new substances exhibiting anti-inflammatory properties holds significant importance for clinical use, with the goal of securing alternative treatments characterized by efficacy and diminished adverse effects. An attractive strategy in this search for new drugs involves the exploration of molecules with anti-inflammatory potential derived from medicinal plants, given the vast and yet undiscovered diversity of bioactive compounds within the plant kingdom [7,8]. Natural products stand out as a noteworthy reservoir for therapeutic interventions against various diseases, with the anti-inflammatory effects of traditional herbal medicines, crude plant extracts, natural compounds, and their derivatives having already demonstrated significant promise [7,9].

In this context, *Solidago chilensis* Meyen (Asteraceae) emerges as a highly promising reservoir for the discovery of anti-inflammatory agents. This plant species, with a historical use spanning over 700 years in South American folk medicine, has been recognized for its diverse therapeutic attributes, including anticancer, antidepressant, diuretic, gastroprotective, burn treatment, and anti-inflammatory activities [10,11,12,13]. 

Phytochemical analyses of *S. chilensis* have revealed a spectrum of chemical compounds, including flavonoids such as quercitrin, quercetin, and rutin, as well as diterpenes such as deoxysolidagenone, solidagolactol, and solidagenone [14]. Notably, the relatively underexplored compound solidagenone, isolated from the rhizomes, leaves, and inflorescences of *S. chilensis*, exhibits promising biological activities [13,15,16,17]. Solidagenone has demonstrated gastroprotective activity in a murine model of gastric injury induced by hydrochloric acid [15], attenuated skin inflammation in experimental models [16], exhibited antidepressant-like effects in mice [13], and a protective effect in a model of airway inflammation induced by ovalbumin [17]. Consequently, solidagenone holds considerable potential for further investigations into its anti-inflammatory activity. The present study was designed to assess the anti-inflammatory properties of solidagenone in acute experimental models of inflammation and its mechanism of action.

## 2. Results and Discussion

Firstly, the toxicity effects of a single dose of solidagenone were investigated. Administration of 30, 60, or 90 mg/kg of solidagenone did not result in mortality or elicit any discernible signs of toxicity in the animals (Table 1). Furthermore, no significant variance in body weight was noted among the animals treated with different doses of solidagenone when compared to mice treated only with the vehicle solution (Table 2).

Toxicological investigations of bioactive compounds derived from medicinal plants are essential to ensure the safety of using herbs in the treatment of a series of diseases [18]. Therefore, it is relevant to analyze the acute toxicity generated by treatment with solidagenone in mice. Here, we verified that the compound did not present toxicity at any of the administered doses used (30, 60, and 90 mg/kg). These findings are consistent with the literature; Rodriguez and collaborators [19] used a similar methodology to the one used in this study and verified that solidagenone, intraperitoneally injected, did not show any observable symptoms of toxicity or mortality in Swiss mice treated with doses ranging from 100 to 600 mg/kg.

A crucial index for evaluating the physiological or pathological state of mice subjected to experimental tests is body weight. Alterations in body weight often correlate with significant physiological changes, warranting a thorough analysis of this parameter [20]. In our study, we found that treatment with solidagenone did not induce significant changes in the weight of the animals.

**Figure 1:**
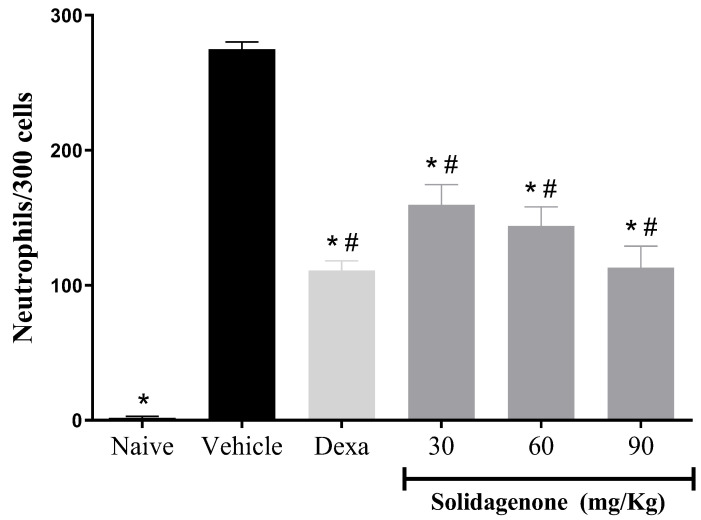
Effect of solidagenone in a model of acute peritonitis. BALB/c mice (*n* = 6/group) were submitted to a challenge with 1% carrageenan solution after treatment with solidagenone (30, 60, and 90 mg/kg) or dexamethasone (Dexa; 30 mg/kg) or vehicle (saline solution with 10% DMSO). Naïve group consisted of untreated and unchallenged animals. Values represent the means ± S.D. of six mice/group. * *p* < 0.05 compared to the vehicle group; # *p* < 0.05 compared to the naïve group.

Next, we assessed the anti-inflammatory activity of solidagenone, first in a mouse model of acute peritonitis induced by carrageenan. As revealed in Figure 1, mice subjected to carrageenan stimulation and treated with a vehicle solution exhibited a mean of 274.8 neutrophils in 300 counted cells, a significant increase (*p* < 0.05) compared to the naïve group which presented a mean of 1.8 neutrophils in 300 counted cells. In comparison to the vehicle-treated group, pre-treatment with solidagenone at doses of 30, 60, and 90 mg/kg resulted in a statistically significant reduction (*p* < 0.05) in neutrophil migration by 58.9%, 47.6%, and 41.9%, respectively. Under the same conditions, the administration of dexamethasone, at a dose of 30 mg/kg, also promoted a significant reduction (*p* < 0.05) of 59.6% in neutrophil migration (Figure 1). In agreement with these data, Liz and colleagues [21] demonstrated that an aqueous rhizome extract of *S. chilensis* and its two derived fractions promoted the inhibition of leukocyte migration, particularly neutrophil migration, and exudation in a mouse model of air pouch using carrageenan. Moreover, these effects were accompanied by a reduction in IL-1β, TNFα, and nitric oxide production, as well as a decrease in myeloperoxidase and adenosine deaminase activity [21]. Using a model of pleurisy induced by carrageenan, similar results were found with extracts from leaves or inflorescences of *S. chilensis* [22].

**Figure 2:**
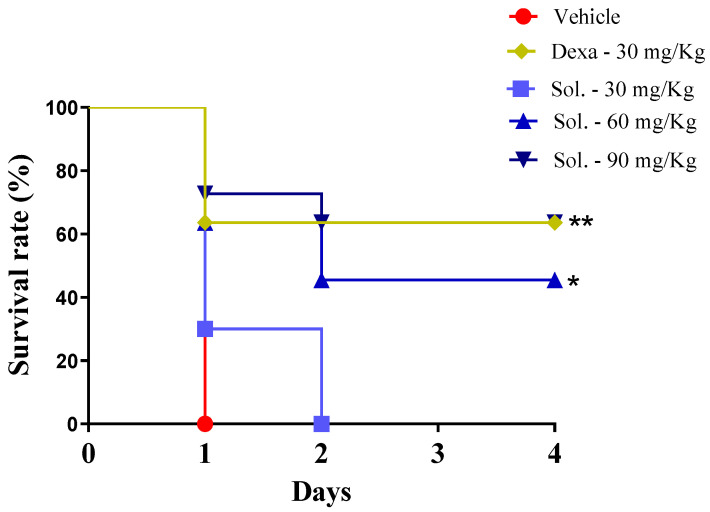
Survival curve of mice treated with solidagenone submitted to endotoxic shock. Mice were orally treated with solidagenone at doses of 30 mg/kg (■), 60 mg/Kg (▲), and 90 mg/kg (▼); dexamethasone at a dose of 30 mg/kg (♦); or vehicle (●). The results are from two experiments performed independently. * *p* < 0.05 compared to the vehicle group. ** *p* < 0.01 compared to the vehicle group. Statistical analyses were performed using the Logrank test (Mantel Cox).

Next, the anti-inflammatory effect of solidagenone was further investigated in a mouse model of endotoxic shock. As revealed in Figure 2, in comparison with the vehicle group, the animals treated with solidagenone at 60 and 90 mg/kg exhibited an increased survival rate of 45.5 and 63.3%, respectively, which was significant (*p* < 0.05). Under the same conditions, dexamethasone, administered at a dose of 30 mg/kg, also promoted a significant increase of 63.3% (*p* < 0.05) in the survival rate.

As expected, LPS administration induced leukopenia and thrombocytopenia in the animals (Table 3). Interestingly, treatment with solidagenone also conferred a significant (*p* < 0.05) protective effect against these hematological disorders, which are commonly observed in sepsis conditions. Dexamethasone, while promoting a more pronounced leukocyte recovery, did not afford protection against thrombocytopenia (Table 3).

**Figure 3:**
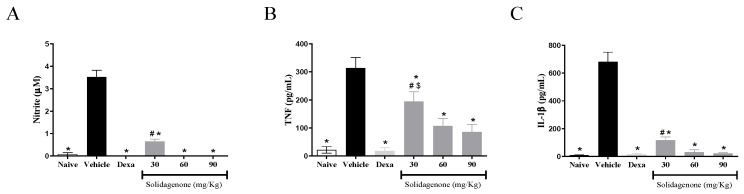
In vivo treatment with solidagenone decreases nitric oxide, TNF-α, and IL-1β production by LPS-stimulated macrophages. Concentrations of nitrite (**A**), TNF-α (**B**), and IL-1β (**C**). Values represent the means ± S.D. of six mice/group. * *p* < 0.05 compared to the vehicle group; # *p* < 0.05 compared to naïve group. $ *p* < 0.05 compared to dexamethasone group.

For a better understanding of the anti-inflammatory effect of solidagenone, we measured the amount of nitric oxide and cytokines in the supernatant of resident macrophages from animals previously treated with solidagenone and stimulated in vitro with LPS plus IFNγ. As revealed in Figure 3, treatment with all the doses of solidagenone promoted a significant reduction in the levels of nitric oxide (Figure 3A), TNF-α (Figure 3B), and IL-1β (Figure 3C) relative to the LPS-stimulated vehicle-treated cultures. In a similar way, dexamethasone, administered at a dose of 30 mg/kg, also promoted a significant (*p* < 0.05) reduction in the levels of all the pro-inflammatory molecules evaluated (Figure 3). In agreement with these data, solidagenone also reduced NO, IL-1β, and TNFα production and gene expression of several inflammatory mediators (*NOS2*, *IL1β*, *TNFα*, and *Cox2*) in peritoneal macrophages stimulated in vitro with LPS plus IFNγ [17]. Moreover, the anti-inflammatory activity of solidagenone was also associated with nitric oxide, IL-6, and TNFα reductions in croton-oil-, arachidonic-acid-, and phenol-induced ear edema mouse models [16].

**Figure 4:**
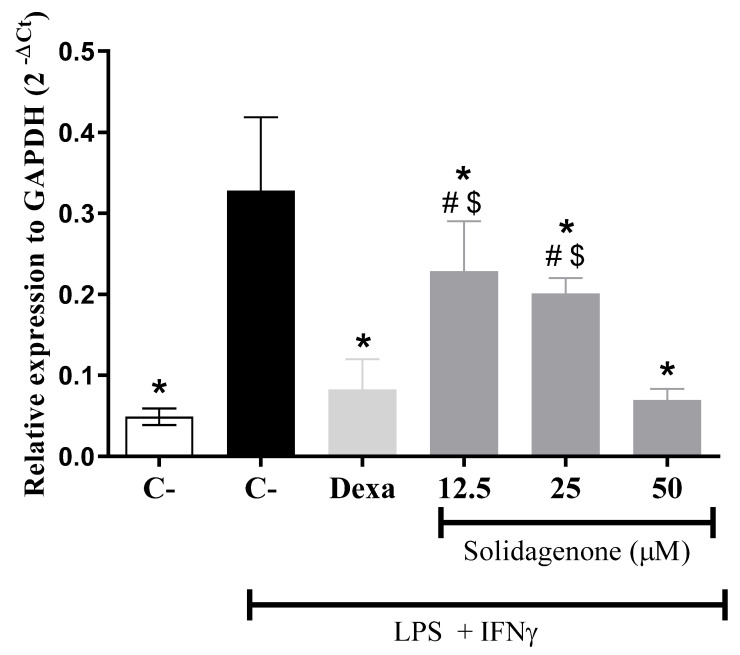
Gene expression of NF-κβ in untreated macrophages or macrophages treated with solidagenone. Values represent the means ± S.D. of four determinations obtained in one of two experiments performed. * *p* < 0.05 compared to stimulated and untreated cells; # *p* < 0.05 compared to unstimulated and untreated cells; $ *p* < 0.05 compared to dexamethasone-treated cells.

It is well established that LPS induces Toll-like receptor-4 (TLR4) activation, triggering intracellular signaling pathways, including NF-κB activation, a pivotal transcription factor that modulates diverse pro-inflammatory genes such as IL-1β, NOS2, and TNF-α [23,24]. Notably, as revealed in Figure 4, the treatment of activated macrophages in vitro with different concentrations of solidagenone results in a significant (*p* < 0.05) reduction in the gene expression of NF-κB. Similar results were obtained with dexamethasone treatment.

To understand the interaction between solidagenone and NF-κB, docking simulations were performed to investigate the interactions between solidagenone and key components involved in the NF-κB signaling pathway: the IκB kinase enzyme (IKK) and the p65 subunit of the NF-κB transcription factor. The NF-κB transcription factor is normally bound to the inhibitor of κB (IκB) in the cytosol, preventing its translocation to the nucleus and the subsequent expression of inflammatory mediators [25,26]. The activation of NF-κB is regulated by the IKK complex, which phosphorylates serine residues on IκB proteins, leading to their polyubiquitination and degradation [27]. This process results in the release of NF-κB, allowing it to translocate to the nucleus and initiate the transcription of genes associated with inflammation [28].

However, the regulation of NF-κB activity extends beyond its liberation from inhibitory subunits and nuclear translocation in the cytoplasm. The transactivation potential of NF-κB in the nucleus, which involves the recruitment of the transcriptional apparatus and the stimulation of target gene expression, is further influenced by post-translational modifications of the transcription factor and its chromatin environment [29]. In this scenario, the p65 subunit of NF-κB was identified as a target of several post-translational modifications such as acetylation, methylation, and phosphorylation [30,31,32,33]. For example, Wei and colleagues [34] demonstrated that the demethylation of arginine 30 (Arg30) in the DNA-binding domain of p65 by protein-arginine methyltransferase 5 (PRMT5) plays a crucial role in the ability of NF-κB to bind to κB elements and regulate gene expression.

Certainly, the p65 subunit of NF-κB and IKK play critical roles in preventing the nuclear translocation and activation of NF-κB. They are involved in different regulatory mechanisms for this nuclear factor, contributing to the control of NF-κB activation [25,34]. In addition, both molecules are widely used as targets for the discovery of new anti-inflammatory drugs, and they are the targets of one of the most used inhibitors of the NF-κB pathway, BAY11-7082 [35,36]. Therefore, docking assays with these proteins are useful in validating new inhibitors of the NF-κB pathway.

**Figure 5:**
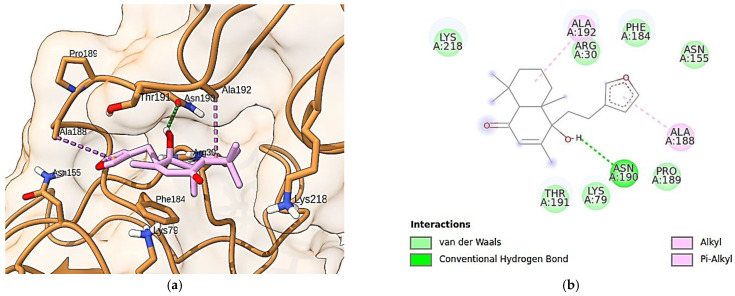
Docking analysis of solidagenone binding to the p65 subunit of NF-κB. (**a**) Residues of the selected protein (PDB ID: 1NFI) and their interactions with solidagenone. (**b**) Two-dimensional interactions of solidagenone with amino acid residues in the p65 subunit of NF-κB.

In this context, we performed docking simulations of solidagenone in the binding site of the p65 subunit of NF-κB (Figure 5). As can be seen, solidagenone docks to the NF-κB system through seven van der Waals interactions (Lys-218, Arg-30, Phe-184, Asn-155, Pro189, Lys-79, and Thr-191), one alkyl interaction (Ala-192), one Pi-alkyl interaction (Ala-188), and one hydrogen bond interaction (Asn-190). The calculated docking energy was equal to −6.4 kcal/mol. Other images of the solidagenone-p65 subunit of NF-κB interaction are shown in Supplementary Material (Figure S1).

**Figure 6:**
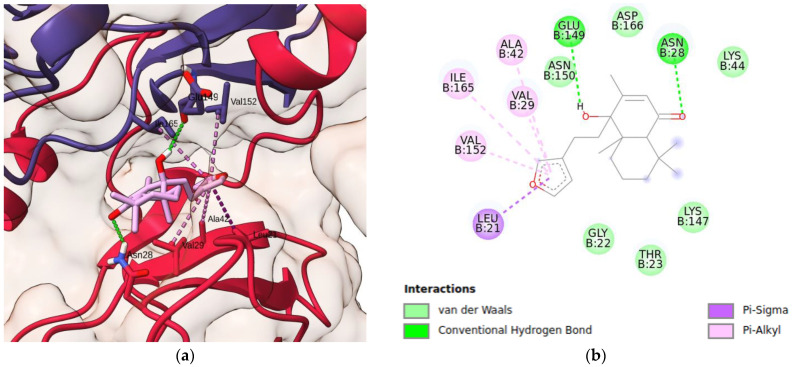
Docking analysis of solidagenone binding to IKK domain. (**a**) Three-dimensional structure of selected protein (PDB ID: 4KIK) and its interaction with solidagenone. (**b**) Two-dimensional interactions of solidagenone with amino acid residues in the IKK domain.

Regarding the IKK system, it is important to mention that it is organized in two chains (A and B), comprising four domains each: the C-terminal dimerization domain (SDD), the C-terminal kinase domain (KDC), the N-terminal kinase domain (KDN), and the ubiquitin-like domain (ULD) (Supplementary Material, Figure S2). Since, according to Liu and coworkers [37], chain B is an active conformation, this was the chain chosen as the protein target for docking solidagenone. Figure 6 shows the details of the simulated IKK–solidagenone complex. Solidagenone docks to the IKK system through six van der Waals interactions (Lys-44, Asp-166, Lys-147, Thr-23, Gly-22, and Asn-150), one Pi-Sigma interaction (Leu-2), four Pi-alkyl interactions (Ala-42, Va-l29, Ile-165, and Val-152), and two hydrogen bond interactions (Asn-28 and Glu-149). The calculated docking energy was equal to −7.8 kcal/mol. Consequently, the solidagenone–IKK interaction is more stable than the solidagenone–NF-κB interaction. Figure S3 shows some images of the solidagenone–IKK interaction. The docking results, as well as the observed reduction in NF-κB gene expression and decreased production of mediators related to NF-κB activation (such as Il-1β, nitric oxide, and TNFα), suggest that the anti-inflammatory effect of solidagenone is linked to the inhibition of the NF-κB pathway. This promising insight provides a foundation for future investigations to delve deeper into the specific mechanisms and interactions involved in the modulation of NF-κB by solidagenone, contributing to a more comprehensive understanding of its anti-inflammatory properties.

**Figure 7:**
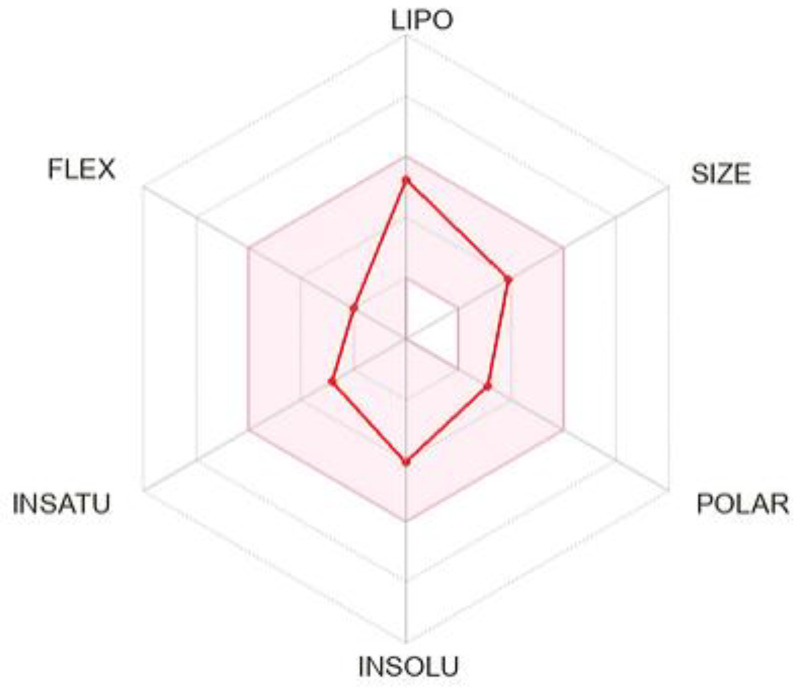
SwissADME plot of drug-likeness of solidagenone. The pink area represents the optimal range for each property. For saturation (INSATU), the ratio of sp^3^ hybridized carbons over the total carbon count of the molecule (Fraction Csp3) should be at least 0.25. For size, the molecular weight should be between 150 and 500 g/mol. For polarity (POLAR), the TPSA should be between 20 and 130 Å. For solubility (INSOLU), log *S* should not exceed 6. For lipophilicity (LIPO), XLOGP3 should be in the range from −0.7 to +6.0. For flexibility (FLEX), the molecule should not have more than 9 rotatable bonds.

Lastly, the in silico ADME profile of solidagenone was generated and is summarized in Figure 7 and Table S1. The *Bioavailability Radar* (Figure 7) has six axes for the six relevant properties for oral bioavailability. Each property has a descriptor, and the range of optimal values is depicted as a pink area. For saturation, the ratio of sp^3^ hybridized carbons over the total carbon count of the molecule (Fraction Csp3) should be at least 0.25. For size, the molecular weight should be between 150 and 500 g/mol. For polarity, the TPSA should be between 20 and 130 Å. For solubility, log *S* should not exceed six. For lipophilicity, XLOGP3 should be in the range from −0.7 to +6.0. For flexibility, the molecule should not have more than nine rotatable bonds. In this context, solidagenone can be estimated to be drug-like, since the red line is fully included in the pink area. Table S1 shows computed parameters for solidagenone, grouped into different sections (physico-chemical properties, lipophilicity, water solubility, pharmacokinetics, druglikeness, and medicinal chemistry).

## 3. Materials and Methods

### 3.1. Drugs

Solidagenone was obtained from *Solidago chilensis* inflorescences from the Pharmaceutical Technology Institute (FarManguinhos, Oswaldo Cruz Foundation, Rio de Janeiro, Brazil), as previously described [16,38]. Dexamethasone (Sigma-Aldrich, St. Louis, MO, USA) was used as positive control in anti-inflammatory experiments. All compounds were solubilized in dimethyl sulfoxide (DMSO; PanReac, Barcelona, Spain) and diluted in culture medium for use in in vitro assays or saline for in vivo assays. The final concentration of DMSO did not exceed 0.1% in all in vitro assays or 10% in all in vivo analyses.

### 3.2. Animals

BALB/c mice (4 to 8 weeks old) were bred and housed at the Gonçalo Moniz Institute (Oswaldo Cruz Foundation, Salvador, Bahia, Brazil) in sterilized cages, under controlled environmental conditions, and provided with a balanced rodent diet and water ad libitum. All animal experiments and procedures were conducted in accordance with the institution’s committee on the ethical handling of laboratory animals and were approved under the number L-IGM-29/2009.

### 3.3. Acute Toxicity in Mice

BALB/c mice (male; 6–8 weeks of age; *n* = 6/group) were divided into four experimental groups and treated orally with a single dose of solidagenone (30, 60, or 90 mg/kg) or vehicle (saline solution with 10% DMSO). Following the completion of treatment, the mice were monitored for general toxicity signs over a 15-day period. This involved the observation of morphological and behavioral changes. Additionally, the body mass of the animals was measured on days 0, 7, and 14.

### 3.4. Induction of Acute Peritonitis in Mice

BALB/c mice (males; 6–8 weeks old) were divided into six experimental groups (*n* = 6) and treated orally with solidagenone (30, 60, or 90 mg/kg), dexamethasone (30 mg/kg), or vehicle (saline solution with 10% DMSO) 24 and 1 h before the challenge. Subsequently, the animals were challenged through an intraperitoneal injection of 250 μL of carrageenan (Sigma-Aldrich, St. Louis, MO, USA; 1 mg/mL; 250 μL), as previously described [39]. After 4 h, the mice were euthanized and peritoneal exudates were harvested by peritoneal lavage using a saline solution. The cells were centrifuged at 400× *g* for 10 min at 4 °C. Cytospin preparations were stained with rapid panoptic and a differential count of 300 cells was performed by a blinded investigator.

### 3.5. LPS-Induced Endotoxin Shock

BALB/c mice (male; 4–5 weeks of age) were randomized into five experimental groups (*n* = 11/group) and treated orally with solidagenone (30, 60, or 90 mg/kg), dexamethasone (30 mg/kg), or vehicle (saline solution with 10% DMSO) 24 and 3 h before the challenge. Subsequently, the animals received a lethal dose of LPS (600 µg; from serotype 0111:B4 *Escherichia coli*, Sigma-Aldrich, St. Louis, MO, USA) in saline via the intraperitoneal route, as previously described [40]. The animals were monitored for 4 days to observe survival. In addition, a second set of experiments was performed, and heparinized blood samples were collected 6 h after the challenge with LPS (600 µg) to analyze the leukocytes and thrombocytes using the PE 7010VET Hematology Analyzer (Shenzhen Prokan Electronics Inc., Shenzhen, China).

### 3.6. Cytokine and Nitric Oxide Production by Resident Macrophages

To evaluate nitric oxide and cytokine production by resident macrophages, groups of male BALB/c mice were orally treated with solidagenone, dexamethasone, or vehicle in the doses described above. After 90 min, the mice were subjected to euthanasia for macrophage collection by means of peritoneal wash using cold Dulbecco’s modified Eagle’s medium (DMEM; Life Technologies, GIBCO-BRL, Gaithersburg, MD, USA). The cells were washed twice with DMEM, resuspended in DMEM supplemented with 10% fetal bovine serum (FBS; GIBCO) and 50 μg/mL of gentamycin (Life Technologies, Carlsbad, CA, USA), and plated into 96-well plates at a density of 2 × 10^5^ cells/well. After 2 h of incubation at 37 °C, the plates were washed with a saline solution and new medium was added to remove non-adherent cells. The cells were then activated with LPS (500 ng/mL) and IFNγ (5 ng/mL) and further incubated at 37 °C and 5% CO_2_. Cell-free supernatants were collected at two different time points after incubation for the quantification of TNFα (4 h) and for the quantification of nitric oxide and IL-1β (24 h) and kept at −80 °C until further use.

### 3.7. Real-Time Reverse Transcription–Polymerase Chain Reaction (qRT-PCR)

Peritoneal exudate macrophages were obtained as previously described [17]. Then, the cells were plated into a 24-well plate at 1 × 10^6^ cells/well in a DMEM medium supplemented with FBS and gentamicin for 24 h at 37 °C and 5% CO_2_. The cells were then pretreated with solidagenone (50, 25, and 12.5 µM) or dexamethasone (12.5 µM) for 1 h and then stimulated with LPS (500 ng/mL) and IFNγ (5 ng/mL) and incubated at 37 °C for 3 h. After treatment, the gene expression of NF-κB was measured as previously described [17]. The following primer sequences were used in real-time PCR assays: *NFkb*:5′-ATGGCAGACGATGATCCCTAC-3 and 3′-TGTTGACAGTGGTATTTCTGGTG-5.

### 3.8. Solidagenone Structure

For molecular modeling purposes, the solidagenone structure was obtained from PubChem, the public chemical database of the National Center for Biotechnology Information (NCBI) of the National Library of Medicine (NLM), an institute of the U.S. National Institutes of Health (NIH, Bethesda, MD, USA) [41]. This structure was energy-minimized using the quantum mechanical modeling method DFT B3LYP/def2-TZVP, which was implemented in the Orca 5.0.3 software [42].

### 3.9. Crystallographic Protein Structures

The crystallographic structures of human proteins p65 subunit of NF-κB, with a resolution of 2.70 Å (PDB ID: 1NFI) [43], and IκB kinase enzyme (IKK), with a resolution of 2.83 Å (PDB ID: 4KIK) [37], were both obtained from the Protein Data Bank [44] and used in the docking simulations with solidagenone. The protein preparation steps were carried out as follows: (1) non-essential water molecules were removed; (2) polar hydrogens were added to the protein; and (3) partial charges were calculated using both the Kollman [45] and Gasteiger’s approaches [46].

### 3.10. Molecular Docking Simulations

Molecular docking simulations were performed with the AutoDock Vina 1.1.2 program [47]. Two molecular graphical programs, UCSF Chimera X [48] and BIOVIA Discovery Studio 2021 [49], were used for visualizing ligand–protein docking interactions in 3D and 2D representations, respectively.

### 3.11. In Silico ADME

The SwissADME platform [50] was used for computing the physicochemical and pharmacokinetics parameters related to the adsorption, distribution, metabolism, and excretion (ADME) properties of solidagenone, as well as its drug-likeness.

### 3.12. Statistical Analyses

Statistical analyses were performed using GraphPad Prism version 8.0 (GraphPad Software, San Diego, CA, USA). One-way analysis of variance (ANOVA) followed by Newman–Keuls multiple comparison tests were used for the comparison of groups. P-values less than 0.05 were considered to indicate statistical significance. The presented data are representative of at least two or three independent experiments.

## 4. Conclusions

Taken together, the findings of the present investigation show that solidagenone exhibits significant anti-inflammatory properties in acute experimental models, potentially through the modulation of the NF-κB signaling pathway. Furthermore, solidagenone shows no acute toxicity, and its in silico ADME profile suggests favorable drug-like characteristics. The presented data fortify the potential of solidagenone as a basis for the development of alternative treatments with reduced adverse effects for inflammatory conditions.

## Figures and Tables

**Table 1:** Effect of solidagenone on behavioral and general appearance of male BALB/c mice.

Behavior or General Appearance	Observations			
	Vehicle	Solidagenone (30 mg/kg)	Solidagenone (60 mg/kg)	Solidagenone (90 mg/kg)
Changes in the eyes	No changes	No changes	No changes	No changes
Changes in the fur	No changes	No changes	No changes	No changes
Changes in the skin	No changes	No changes	No changes	No changes
Coma	Absent	Absent	Absent	Absent
Convulsions	Absent	Absent	Absent	Absent
Diarrhea	Absent	Absent	Absent	Absent
Lethargy	Absent	Absent	Absent	Absent
Salivation	Absent	Absent	Absent	Absent
Sleep	Usual	Usual	Usual	Usual
Tremors	Absent	Absent	Absent	Absent

The animals were subjected to daily observations for a duration of 14 days.

**Table 2:** Body weight of BALB/c mice treated with compound solidagenone.

Days	Vehicle	Solidagenone (30 mg/kg)	Solidagenone (60 mg/kg)	Solidagenone (90 mg/kg)
0	24.3 (±0.6)	24.1 (±0.3)	23.8 (±1.6)	22.8 (±0.9)
7	24.2 (±0.3)	24.7 (±0.5)	24.0 (±1.8)	22.8 (±1.2)
14	24.4 (±0.5)	24.8 (±0.6)	24.5 (±1.5)	23.0 (±0.7)

Values represent the mean ± standard deviation of six animals per group.

**Table 3:** Solidagenone attenuates leukopenia and thrombocytopenia in mice challenged with LPS.

Group	Dose (mg/kg)	Leukocytes (10^3^ cells/µL)	Thrombocytes (10^3^ mm^3^)
Naive	-	3.8 ± 0.9 *	449.8 ± 49.2 *
Vehicle	-	1.7 ± 0.2	334.5 ± 36.7
Dexamethasone	30	3.8 ± 0.4 *	337.0 ± 26.8
Solidagenone	30	1.9 ± 0.3	365.8 ± 15.2
Solidagenone	60	2.2 ± 0.4	399.4 ± 3.4 *
Solidagenone	90	2.7 ± 0.5 *	403.5 ± 40.4 *

Values represent the mean ± standard deviation of six animals per group. * *p* < 0.05 compared to the vehicle group.

## Data Availability

The data presented in this study are available on request from the corresponding author.

## Supplementary Materials

The following supporting information can be downloaded at: https://www.mdpi.com/article/10.3390/ph17030273/s1, Figure S1: Solidagenone docked onto the p65 subunit of NF-κB; Figure S2: IKK system; Figure S3: IKK system and details of solidagenone–IKK interaction. Table S1: ADME parameters, drug-likeness, and medicinal chemistry of solidagenone.
