# Supplementary material for: Solidagenone from Solidago chilensis Meyen Protects against Acute Peritonitis and Lipopolysaccharide-Induced Shock by Regulating NF-κB Signaling Pathway
Source: Pharmaceuticals (Basel). 2024 Feb 21;17(3):273. doi: 10.3390/ph17030273 (PMC10974014; doi:10.3390/ph17030273)
Supplement: Supplementary file 1 [file pharmaceuticals-17-00273-s001.zip › pharmaceuticals-2822762-supplementary.pdf]

Supplementary Information

Solidagenone from *Solidago chilensis* Meyen protects against acute peritonitis and lipopolysaccharide-induced shock by regulating the NF-κB signaling pathway

Ivanilson Pimenta Santos[1], Laís Peres Silva[1], Dahara Keyse Carvalho Silva[1], Bruna Padilha Zurita Claro dos Reis[1], Temistocles Barroso de Oliveira[2], Andressa Maia Kelly[2], Edivaldo dos Santos Rodrigues[3], Claudia Valeria Campos de Souza[3], José Fernando Oliveira-Costa[4], Simone Sacramento Valverde[2], Osvaldo Andrade Santos-Filho[3], Milena Botelho Pereira Soares[1,5,*], and Cássio Santana Meira[1,5,*]

[1] Gonçalo Moniz Institute, Oswaldo Cruz Foundation, FIOCRUZ, Salvador, BA, 40296-710, Brazil;
[2] Pharmaceutical Technology Institute - FarManguinhos, Natural Products Department, Oswaldo Cruz Foundation, FIOCRUZ, Rio de Janeiro, RJ, 21041-250, Brazil;
[3] Molecular Modeling and Computational Structural Biology Laboratory, IPPN, Health Sciences Center, Federal University of Rio de Janeiro, RJ, 21941-599, Brazil;
[4] Center for Infusions and Specialized Medicines of Bahia, Bahia State Health Department, Salvador, BA, 41745-900, Brazil;
[5] Institute of Innovation in Advanced Health Systems (ISI SAS), University Center SENAI/CIMATEC, Salvador, BA, 41650-010 Brazil.
*Correspondence: cassio.meira@fieb.org.br; milena@bahia.fiocruz.br

Supplementary Information

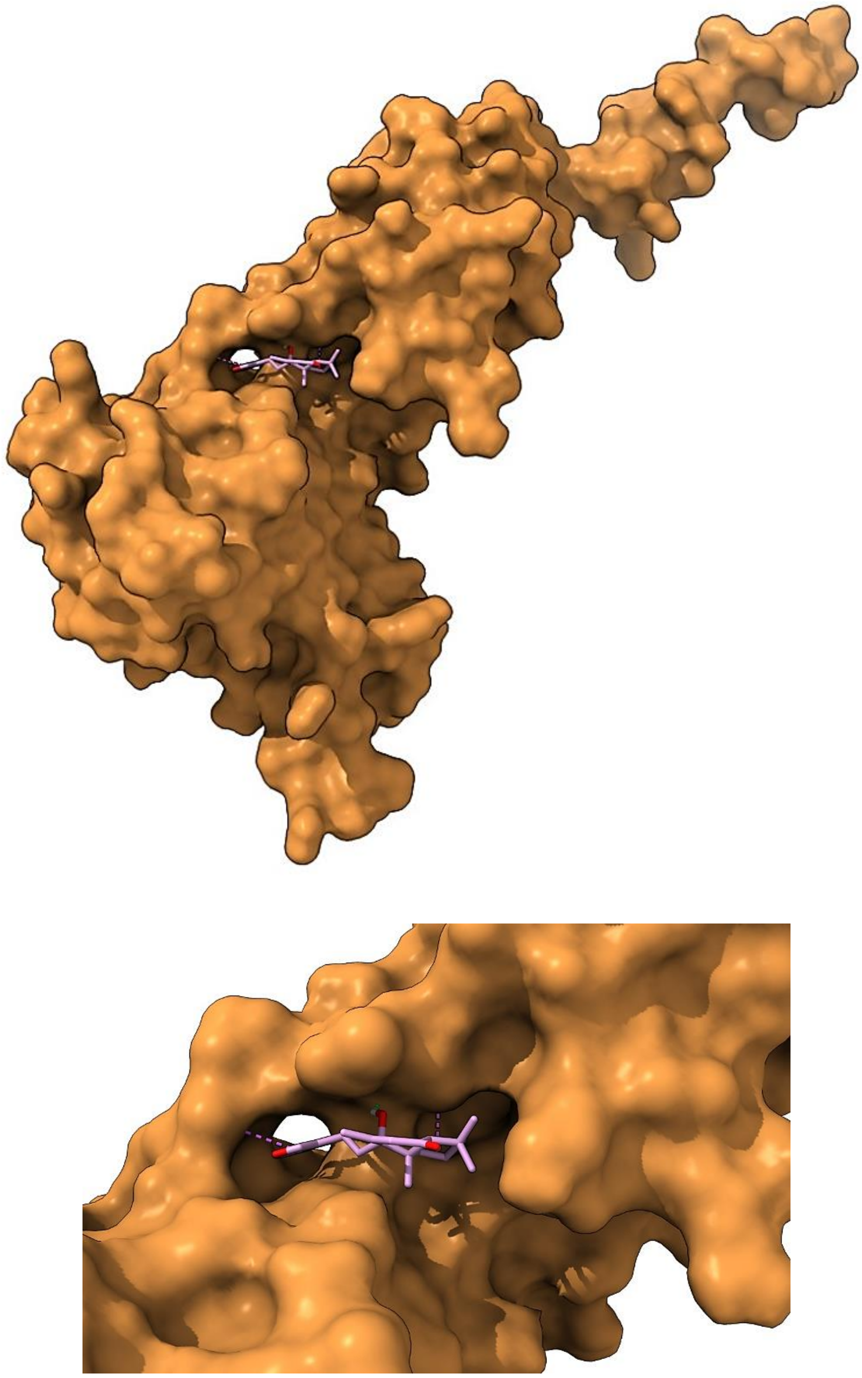

Figure S1. Solidagenone docked into the p65 subunit of NF-κB.

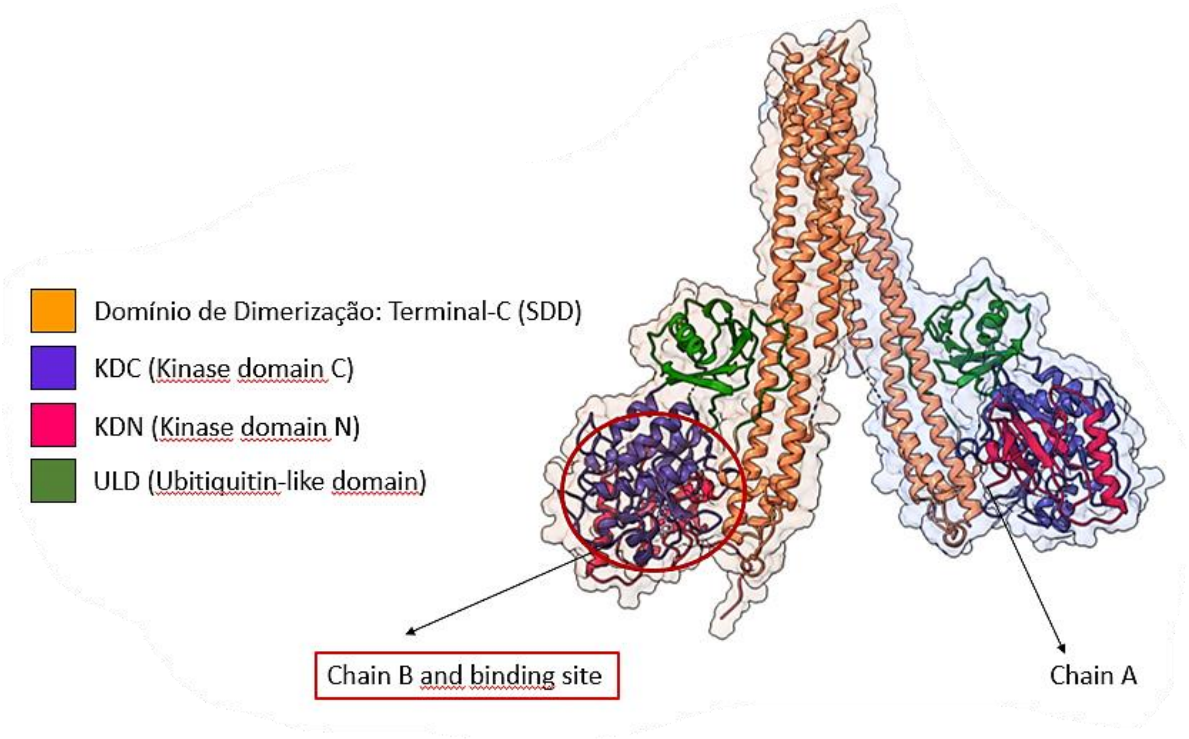

Figure S2. IKK system.

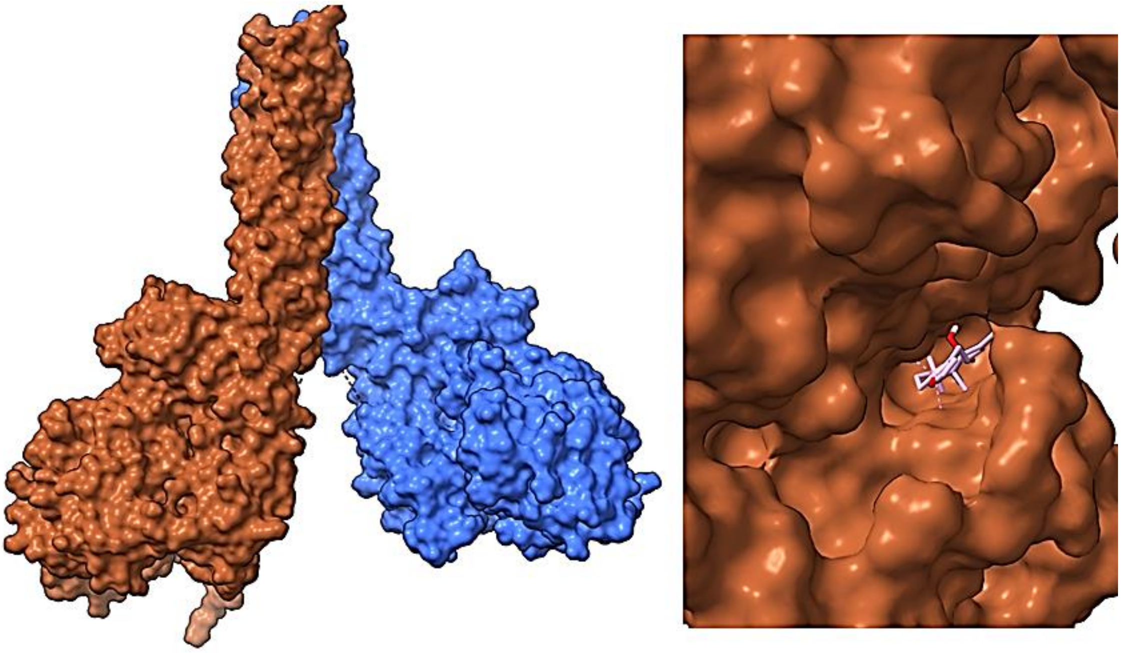

Figure S3. IKK system, and detail of solidagenone-IKK interaction.

Table S1. ADME parameters, druglikeness, and medicinal chemistry of solidagenone.

Physicochemical Properties	
Formula	$C_{20}H_{28}O_3$
Molecular weight	316.43 g/mol
Num. heavy atoms	23
Num. arom. heavy atoms	5
Fraction Csp3	0.65
Num. rotatable bonds	3
Num. H-bond acceptors	3
Num. H-bond donors	1
Molar Refractivity	91.96
TPSA	50.44 Å^2
**Lipophilicity**	
Log $P_{o/w}$ (iLOGP)	3.29
Log $P_{o/w}$ (XLOGP3)	3.63
Log $P_{o/w}$ (WLOGP)	4.30
Log $P_{o/w}$ (MLOGP)	2.71
Log $P_{o/w}$ (SILICOS-IT)	4.61
Consensus Log $P_{o/w}$	3.71
**Water Solubility**	
Log *S* (ESOL)	-4.05
Solubility	2.81e-02 mg/ml; 8.88e-05 mol/l
Class	Moderately soluble
Log *S* (Ali)	-4.38
Solubility	1.33e-02 mg/ml; 4.19e-05 mol/l
Class	Moderately soluble
Log *S* (SILICOS-IT)	-5.53
Solubility	9.40e-04 mg/ml; 2.97e-06 mol/l
Class	Moderately soluble
**Pharmacokinetics**	
GI absorption	High
BBB permeant	Yes
P-gp substrate	Yes
CYP1A2 inhibitor	No
CYP2C19 inhibitor	No
CYP2C9 inhibitor	No
CYP2D6 inhibitor	Yes
CYP3A4 inhibitor	Yes
Log K_P (skin permeation)	-5.65 cm/s
**Druglikeness**	
Lipinski	Yes; 0 violation

Ghose	Yes
Veber	Yes
Egan	Yes
Muegge	Yes
Bioavailability Score	0.55
**Medicinal Chemistry**	
PAINS	0 alert
Brenk	0 alert
Leadlikeness	No; 1 violation: XLOGP3>3.5
Synthetic accessibility	4.61
